# L-arginine Supplementation Improves Responses to Injury and Inflammation in Dextran Sulfate Sodium Colitis

**DOI:** 10.1371/journal.pone.0033546

**Published:** 2012-03-12

**Authors:** Lori A. Coburn, Xue Gong, Kshipra Singh, Mohammad Asim, Brooks P. Scull, Margaret M. Allaman, Christopher S. Williams, Michael J. Rosen, M. Kay Washington, Daniel P. Barry, M. Blanca Piazuelo, Robert A. Casero, Rupesh Chaturvedi, Zhongming Zhao, Keith T. Wilson

**Affiliations:** 1 Division of Gastroenterology, Hepatology, and Nutrition, Department of Medicine, Vanderbilt University Medical Center, Nashville, Tennessee, United States of America; 2 Department of Pathology, Microbiology, and Immunology, Vanderbilt University Medical Center, Nashville, Tennessee, United States of America; 3 Department of Cancer Biology, Vanderbilt University Medical Center, Nashville, Tennessee, United States of America; 4 Department of Biomedical Informatics, Vanderbilt University Medical Center, Nashville, Tennessee, United States of America; 5 Veterans Affairs Tennessee Valley Healthcare System, Nashville, Tennessee, United States of America; 6 Department of Oncology, Sidney Kimmel Comprehensive Cancer Center, Johns Hopkins University School of Medicine, Baltimore, Maryland, United States of America; 7 D. Brent Polk Division of Pediatric Gastroenterology, Department of Pediatrics, Vanderbilt University Medical Center, Nashville, Tennessee, United States of America; Charité-Universitätsmedizin Berlin, Germany

## Abstract

Inflammatory bowel disease (IBD), consisting of Crohn's disease and ulcerative colitis (UC), results in substantial morbidity and is difficult to treat. New strategies for adjunct therapies are needed. One candidate is the semi-essential amino acid, L-arginine (L-Arg), a complementary medicine purported to be an enhancer of immunity and vitality in the lay media. Using dextran sulfate sodium (DSS) as a murine colonic injury and repair model with similarities to human UC, we assessed the effect of L-Arg, as DSS induced increases in colonic expression of the *y* ^+^ cationic amino acid transporter 2 (CAT2) and L-Arg uptake. L-Arg supplementation improved the clinical parameters of survival, body weight loss, and colon weight, and reduced colonic permeability and the number of myeloperoxidase-positive neutrophils in DSS colitis. Luminex-based multi-analyte profiling demonstrated that there was a marked reduction in proinflammatory cytokine and chemokine expression with L-Arg treatment. Genomic analysis by microarray demonstrated that DSS-treated mice supplemented with L-Arg clustered more closely with mice not exposed to DSS than to those receiving DSS alone, and revealed that multiple genes that were upregulated or downregulated with DSS alone exhibited normalization of expression with L-Arg supplementation. Additionally, L-Arg treatment of mice with DSS colitis resulted in increased *ex vivo* migration of colonic epithelial cells, suggestive of increased capacity for wound repair. Because CAT2 induction was sustained during L-Arg treatment and inducible nitric oxide (NO) synthase (iNOS) requires uptake of L-Arg for generation of NO, we tested the effect of L-Arg in iNOS^−/−^ mice and found that its benefits in DSS colitis were eliminated. These preclinical studies indicate that L-Arg supplementation could be a potential therapy for IBD, and that one mechanism of action may be functional enhancement of iNOS activity.

## Introduction

Inflammatory bowel disease (IBD) is postulated to arise from the interplay between luminal bacteria and the colonic mucosa [1], [2]. There are two major forms: ulcerative colitis (UC) and Crohn's disease and approximately 1.4 million Americans are affected [1], [2]. The mechanisms underlying the abnormal immune system response continue to be investigated with the hope that new therapeutics may arise from a better understanding of pathophysiology. There is growing interest by patients with diseases such as IBD in dietary supplements including macronutrient. One such supplement that has been extensively promoted in the lay media for enhancement of immunity and for use in a variety of health concerns is L-arginine (L-Arg), and it is therefore used orally as a complementary/alternative medicine. L-Arg is a semi-essential amino acid that is important in protein synthesis. Because L-Arg can be synthesized by the body, dietary intake of arginine is not essential to maintain nitrogen balance in normal adults [3]. However, total body L-Arg may become depleted under stressful conditions [4], [5].

Cellular uptake of L-Arg is an active transport process mediated primarily by cationic amino acid transporter (CAT) family proteins, also known as *y* ^+^ transporters [6]. CAT proteins 1–4 have been described. CAT2 is the inducible transporter, and is known to be upregulated in macrophages upon immune activation [7], [8], while CAT1 is constitutively expressed [8]. The potential importance of CAT3 and CAT4 is poorly understood [9], [10]. CAT proteins, in particular, are subject to competitive inhibition by L-ornithine (L-Orn) and L-lysine (L-Lys) [9], [10], [11]. L-Arg is a substrate for four enzymes, some of which exist as multiple isoforms: arginase, nitric oxide (NO) synthase (NOS), arginine: glycine amidinotransferase, and arginine decarboxylase [12]. Arginase enzymes are the endogenous antagonists to inducible NOS (iNOS) because they compete for the same L-Arg substrate by metabolizing it to L-Orn and urea [13], whereas iNOS metabolizes L-Arg to NO and L-citrulline (L-Cit). There are two isoforms of arginase: arginase I (Arg1) is abundant in liver and is important for the urea cycle, and arginase II (Arg2) is abundant in kidney and localizes to mitochondria [14], [15]. L-Orn is used by ornithine decarboxylase (ODC) to produce the polyamine putrescine, which is then converted into the polyamines spermidine and spermine by constitutively expressed spermidine and spermine synthases, respectively. Notably, polyamines have been associated with mucosal protection in the gastrointestinal tract [16], [17] including intestinal epithelial cell migration [18]. L-Orn can also be acted upon by ornithine aminotransferase (OAT) to produce L-proline (L-Pro) [19], [20]. L-Pro is an important precursor in collagen synthesis and has been demonstrated to be involved in wound healing and cell migration in fibroblasts [21], and in bovine retinal pigment epithelial cells [22].

There is literature suggesting that L-Arg metabolism in the alimentary tract is altered in animal models of ischemic colitis and inflammatory bowel disease [23]. In a rat model, L-Arg supplementation attenuated the degree of tissue damage in intestinal ischemia and promoted healing of intestinal mucosa [24]. We have previously shown that in the *Citrobacter rodentium* infection model of murine colitis there is a significant decrease in the serum L-Arg concentration versus control mice, and that L-Arg supplementation is clinically beneficial while inhibition of polyamine synthesis is deleterious [25]. We have also reported increased serum concentrations of L-Arg in patients with severe UC, but relative arginine availability [26] was not increased in these subjects due to the concomitant increase in serum L-Orn and L-Lys [27], the competitive inhibitors for L-Arg transport [10], [11], [27].

Although the precise mechanisms underlying the inflammation and immune responses in IBD are still being investigated, various inflammatory mediators, including proinflammatory cytokines have been implicated in the disease process [28]. In particular, increased secretion of proinflammatory cytokines is thought to be important for exacerbation of IBD. A number of therapeutic approaches targeting these factors are available. Administration of an anti-TNF-α antibody in mice [29], [30] and in humans (Infliximab) has demonstrated efficacy [31], [32], [33], but these treatments are often complicated by significant adverse effects and expense. Additionally, anti-TNF-α therapies have limited effectiveness with a therapeutic response in only about half of patients [34], indicating the need to target more than just TNF signaling.

Therefore, ongoing research into new treatment approaches, including micronutrient supplements, may be useful. For example, in our previous study in *C. rodentium* colitis in mice, L-Arg supplementation led to decreased proinflammatory cytokine mRNA levels [25]. This led to our goal of assessing the role of L-Arg in more depth in the regulation of injury and inflammation in a model of experimental colitis that has dysfunction of epithelial integrity and immune dysregulation. Dextran sulfate sodium (DSS) is a heparin-like polysaccharide that has been successfully used to induce colonic mucosal injury in mice [35]. We selected this model for the current study because DSS-induced colitis exhibits characteristics resembling human UC, including weight loss, severe diarrhea, rectal bleeding, loss of epithelium followed by ulceration and leukocyte infiltration [35], [36]. DSS has been linked to direct epithelial cytotoxicity and interference with the normal interaction between intestinal lymphocytes and epithelial cells [35], [37], [38].

We now report that in the DSS murine model of colonic injury, oral L-Arg treatment improves clinical parameters as well as proinflammatory cytokine and chemokine responses. Furthermore, we show a reduction in the tissue myeloperoxidase-positive inflammatory cell infiltrate, improved mucosal integrity, and enhanced epithelial cell migration. Genomic analysis by microarray revealed multiple differentially expressed genes after exposure to DSS and many of these changes were abrogated with L-Arg supplementation. Similarly, Luminex-based profiling revealed that multiple proinflammatory cytokines and chemokines upregulated by DSS were restored to normal levels with L-Arg treatment. We also show that iNOS-deficient mice exhibited loss of the salutary effects of L-Arg, implicating the iNOS/NO pathway in the healing phase of DSS colonic injury.

## Materials and Methods

### Ethics statement

This study was carried out following recommendations in the Guide for the Care and Use of Laboratory Animals of the National Institutes of Health. The protocol was approved by the Institutional Animal Care and Use Committee of Vanderbilt University (Protocol Number M/08/124). The study was also approved by the Research and Development Committee of the Veterans Affairs Tennessee Valley Healthcare System.

### Animals

iNOS^−/−^ C57BL/6 mice were originally obtained from The Jackson Laboratory (Bar Harbor, ME) and bred in our facility. Six-week-old wild-type (WT) C57BL/6 mice were purchased from The Jackson Laboratory. Only male mice were used for experiments, and all mice were used at 7 weeks of age. Animals were maintained on a 12 h light/12 h dark cycle under biosafety level 2 conditions. The mice had *ad libitum* access to a standard diet and water until reaching the desired age for experiments.

### Induction of DSS colitis

DSS (MW 36,000–50,000, MP Biomedical, Solon, OH) was added to the drinking water of the mice [39] for 6 or 7 days, as indicated in the Results. The final concentration was 4% (wt/vol). Mice were allowed free access to water during the experiment. On the day of sacrifice, the colons were removed, and after the length was measured, they were rinsed clean, blotted dry, weighed and Swiss-rolled for histology. In addition, two proximal and two distal 2 mm^2^ samples were reserved from each colon, and one from each region was combined. Of these, one set of samples was placed in RNAlater® (Ambion, Austin, TX) for RNA analysis. The second set was snap-frozen and used for protein analysis or amino acid profiling.

### L-arginine

In all *in vivo* experiments, L-Arg (Sigma-Aldrich, St. Louis, MO, catalogue number A8094) was administered as a 1% (wt/vol) solution in the drinking water. L-Arg was used for 4 days after 6 days of DSS.

### Survival and body weight measurement

To assess the effect of DSS treatment on the mice, survival and changes in body weight of the animals were monitored daily over the course of colitis development. Mice were monitored throughout the experiment and any that showed extreme distress, became moribund, or lost more than 20% of initial body weight were euthanized.

### Assessment of histological score

Swiss-rolled colons were formalin-fixed and paraffin-embedded, and 5 µm sections were stained with hematoxylin and eosin and examined in a blinded manner by a gastrointestinal pathologist (MKW). Tissues were scored on a 0–40 scale based on the parameters of inflammation severity (0–3), inflammation extent (0–3), and crypt damage (0–4) each multiplied by the percent involvement (1 = 0–25%; 2 = 25–50%; 3 = 50–75%; and 4 = 75–100%) as described previously [36], [40], [41].

### mRNA analysis

mRNA was isolated as described [36]. Then, 1 µg of RNA was reverse-transcribed using an iScript cDNA synthesis kit (Bio-Rad, Hercules, CA). Each PCR reaction was performed with 2 µl of cDNA and 2× LightCycler® 480 SYBR Green I Master Mix (Roche, Indianapolis, IN). Primers for β-actin were used as described [25], [42]. Primers for CAT1 [43], [44], [45], CAT2 [43], [44], [45], Arg1 [14], Arg2 [14], iNOS [42], and ODC [45], [46] were used as previously described. The sequences for OAT primers were as follows: (F) 5′ ACCATTGCTGCTCTGCTGCGCCG 3′, and (R) 5′ TGAAGTACTGCCTGCCTTCCACA 3′. The thermal cycling conditions and the method used to calculate relative expression have been described previously [44], [45], [46].

### Western blot analysis

Samples of frozen colon tissue were suspended in CelLytic™ MT Mammalian Tissue Lysis/Extraction Reagent (Sigma) containing EDTA-free Protease Inhibitor Cocktail Set III, and Phosphatase Inhibitor Set I (EMD Chemicals) and disrupted by three 10-second pulses of sonication at 40 W (Ultrasonic Processor GE 130PB, Hielscher). For each mouse, 80 µg of protein was resolved per lane on a 10% polyacrylamide gel (Bio-Rad) and transferred overnight onto PVDF. After blocking with 5% milk for 2 h, CAT1, CAT2, iNOS, Arg1, Arg2, ODC, and β-actin were detected by Western blotting as described [47]. CAT1 (67 kDa), CAT2 (80 kDa), iNOS (130 kDa), Arg1 (38 kDa), Arg2 (40 kDa), ODC (53 kDa), and β-actin (42 kDa) proteins were detected with the following antibodies: rabbit polyclonal CAT1 and CAT2 Abs (1/500; both from E. I. Closs, Johannes Gutenberg University, Mainz, Germany); rabbit polyclonal iNOS Ab (1/2000; BD Biosciences); rabbit polyclonal Arg1 Ab (1/200; Santa Cruz Biotechnology, Santa Cruz, CA); goat polyclonal Arg2 Ab (1/200; Santa Cruz Biotechnology, Santa Cruz, CA); goat polyclonal ODC Ab (1/500; Santa Cruz Biotechnology, Santa Cruz, CA); and mouse polyclonal β-actin Ab (1/10000; Sigma).

### Assessment of serum and tissue amino acid concentrations

Blood was collected at the time of sacrifice via cardiac puncture and placed in an empty microcentrifuge tube. The samples were placed on ice and were processed at 30 min. The samples were centrifuged for 10 min at 13,400×*g*. The supernatant was removed, aliquoted, snap frozen, and stored at −80°C until used for amino acid analysis.

Frozen colonic tissue (20–30 mg) was lysed in 250 µL of a 10% 5-sulfosalicylic acid solution (SSA) (wt/vol) with a mortar and pestle-type rotary homogenizer. The precipitated proteins were pelleted via centrifugation at 13,400×*g* for 10 min and the supernatant removed, snap frozen, and stored at −80°C for subsequent amino acid analysis. Protein concentration was measured in the lysates by the bicinchoninic acid (BCA) method as described [43].

Serum samples and tissue supernatants were provided to the Vanderbilt Hormone Assay Core for amino acid analysis via high performance liquid chromatography (HPLC). A dedicated Biochrom (Holliston, MA) 30 Amino Acid Analyzer equipped with an autosampler was used for the determination of amino acid concentrations. Serum samples were deproteinized with an equal volume of 10% SSA containing norleucine, which was added as an internal standard. Tissue supernatants were diluted 1∶1 with loading buffer containing norleucine. Amino acid separation was achieved utilizing a lithium citrate buffering system on an ion exchange column, followed with a ninhydrin post column derivatization at 135**°**C, and photometric detection. The peak areas at 440 nm and 570 nm were integrated using EZChrom software (Agilent Technologies, Santa Clara, CA).

The relative L-Arg availability was assessed in serum and tissues. This was determined by the arginine availability index (AAI) [27], calculated as [L-Arg]/([L-Lys]+[L-Orn]).

### Tissue L-arginine uptake

The uptake studies were initiated by adding 10 µL of [^14^C]-L-Arg (specific activity, 346 mCi/mmol) to the tissue for 5 min in phosphate-buffered saline (PBS). The tissue was then washed 3 times with cold PBS, and then lysed in 250 µL of radioimmunassay precipitation assay (RIPA) buffer with a mortar and pestle-type rotary homogenizer. The lysates were centrifuged for 20 min at 9,300×*g* and supernatants were collected. Then, 10 µL of the supernatant was added to 5 mL of scintillation fluid and the ^14^C content was determined in a scintillation counter. Protein concentration was measured in the lysate using the BCA method [43]. L-Arg transport values were expressed as pmol [^14^C]-L-Arg/min/mg protein.

### Assessment of colonic mucosal permeability

Two hours prior to sacrifice, mice were anesthetized with inhaled, 5% isofluorane and given 200 µL of an 80 mg/mL solution of fluorescein isothiocyanate (FITC)–dextran (Sigma Aldrich, St. Louis, MO) via rectum using a 1 mL syringe with a modified neonatal feeding tube. At the time of sacrifice, blood was obtained via cardiac puncture, placed in an empty microfuge tube, and placed on ice. The samples were centrifuged (9,300×*g* at 4°C) for 20 min. Serum (50 µL)was mixed with an equal volume of PBS and added to a black 96-well plate. The amount of fluorescence was determined by spectrophotofluorometry with an excitation wavelength of 482 nm and an emission wavelength of 528 nm using serially diluted samples of the marker as a standard in a BioTek (Winooski, VT) Synergy 4 plate reader.

### Immunohistochemistry for myeloperoxidase

Formalin-fixed and paraffin-embedded 5 µm sections of Swiss-rolled colons were stained with myeloperoxidase (MPO) prediluted polyclonal antibody (Biocare Medical, Concord, CA) per the manufacturer's protocols. Subsequently, the slides were incubated for 5 min at room temperature with 3,3′-diaminobenzidine (Biocare Medical, Concord, CA). The slides were examined in a blinded manner by a gastrointestinal pathologist (MBP). Tissues were scored as the number of MPO-positive cells per high-power field (HPF). A total of 20 HPFs were assessed for each section. The scores were then averaged for each section.

### Assessment of tissue polyamine concentrations

Frozen colonic tissue (20–30 mg) was lysed in 500 µL RIPA buffer with a mortar and pestle-type rotary homogenizer. Protein concentration was measured in the lysate, using the BCA method [43]. Polyamine levels in the tissue samples were determined by precolumn dansylation reverse phase HPLC as reported previously [25], [43], [45].

### Tissue cytokine and chemokine analysis

Colon tissues were reserved for protein analysis as described above. The tissues were lysed in RIPA buffer with a mortar and pestle-type rotary homogenizer, and used for Luminex assay as described [48]. The lysates were then assessed by a BioPlex® (Bio-Rad, Hercules, CA) multiplex bead-based antibody detection kit according to the manufacturer's protocols [49]. Protein concentration was measured in the lysate using the BCA method as described [43].

### Microarray assessment

RNA samples were prepared from colon tissues as described above. Each sample was quantified on the Nanodrop ND-1000 Spectrophotometer (ThermoFisher Scientific, Willmington, DE) according to the manufacturer's protocols and as described in Supplemental Methods (Methods S1). Sample integrity was assessed (Methods S1) using an Agilent 2100 Bioanalyzer (Agilent Technologies, Santa Clara, CA) according to the manufacturer's protocols. All of our samples had an RNA Integrity Number (RIN) of >9 (scale 0–10). The reactions, hybridization, and data processing were performed in the Vanderbilt Genome Sciences Resource using the Ambion Whole Transcript Reaction kit following the manufacturer's protocol. Three biologic replicates were profiled for each treatment group. The microarray data were normalized using the robust multichip average (RMA) method [50], [51]. Additional details about the microarray techniques are provided in Methods S1.

### Analysis of microarray results

Hierarchical clustering of global gene expression patterns was performed. First, the 12 total samples were clustered using complete linkage analysis by the expression values of the probes to examine the inter- and intra-group relationships. This information was used to visually display the relationships based on all the probes present. Differentially expressed genes (DEGs) were selected by both a *p* value<0.01 based on Significance Analysis of Microarrays (SAM) [50], [52], and fold change >2. An ANOVA analysis was performed to identify the DEGs among the control, L-Arg, DSS, and DSS+L-Arg groups. To identify groups of genes induced or downregulated by DSS that were then altered by L-Arg supplementation, hierarchical clustering using a Pearson correlation as the similarity metric of the mean of each group was performed. Based on the gene expression values, four clusters were characterized from the hierarchical dendrogram. From this clustering, we were able to identify 4 distinct gene expression patterns. The functional features of the DEGs were evaluated using Ingenuity Pathway Analysis. The microarray data follow MIAME requirements and have been deposited in the NCBI Gene Expression Omnibus (GEO) database and can be accessed at the following link (http://www.ncbi.nlm.nih.gov/geo/query/acc.cgi?token=btwzpmaqccysany&acc=GSE34874).

### Confirmation of microarray results by PCR

Confirmation of the gene expression values was performed on the same RNA samples that were used for the microarray analysis. We chose 4 genes to test. mRNA was isolated as previously described. Then, 1 µg of RNA was reverse-transcribed using an iScript cDNA synthesis kit (Bio-Rad, Hercules, CA). Each PCR reaction was performed with 2 µL of cDNA and 2× LightCycler® 480 SYBR Green I Master Mix (Roche, Indianapolis, IN). Primers for β-actin were used as described [25], [42]. The sense and anti-sense primer sequences were as follows: MMP-3 5′-GAAGAAGATCGATGCTGCCA-3′ and 5′-AGATCCACTGAAGAAGTAGAG-3′, MMP-10 5′-GCTGTTTTTGAAAAGGAGAAGAA-3′ and 5′-AGTATGTGTGTCACCGTCCT-3′, MMP-13 5′-GCGGTTCACTTTGAGAACAC-3′ and 5′-GCATGACTCTCACAATGCGA-3′, and IL-6 5′-TCCACGATTTCCCAGAGAAC-3′ and 5′-AGTTGCCTTCTTGGGACTGA-3′. The thermal cycling conditions and the method used to calculate relative expression have been described previously [44], [45], [46].

### Assessment of epithelial proliferation

Mice were given an intraperitoneal injection of 5-bromo-2′-deoxyuridine (BrdU; 150 µL of a 10 mg/mL solution) 90 min prior to sacrifice. The colon was obtained for epithelial cell isolation and staining for E-cadherin as an epithelial cell marker as previously described [36]. The isolated epithelial cells were then stained for BrdU using the APC BrdU Flow Kit (BD Biosciences, San Jose, CA) per the manufacturer's protocols. The percentage of BrdU positive, E-cadherin positive cells was assessed by flow cytometry.

### Epithelial migration assay

Colonic epithelial cells were isolated as above. Migration of freshly isolated mouse colonic epithelial cells was measured using a migration kit from Chemicon International (Millipore, Billerica, MA; QCM Chemotaxis 8 µm 96-well Cell Migration Assay), according to the manufacturer's instructions. Briefly, 2.5×10^4^ cells were plated in 96-well migration chambers. L-Arg (1.6 mM) was added to the medium in the feeder trays underlying the cell chambers, as well as in the migration chambers. Plates were incubated for 24 h at 37°C in a CO_2_ incubator. Cells and medium from the top side of the migration chambers were discarded and the migration chamber plates were placed on top of new 96-well feeder trays containing 150 µL of cell detachment solution, and incubated for 30 min at 37°C. CyQuant GR Dye was diluted with lysis buffer and added to each well of the feeder trays, and incubated for 15 min at room temperature. 150 µL of the mixture from the feeder trays was transferred to new black 96-well plates and fluorescence was measured at 480 nm and 520 nm in a BioTek Synergy 4 plate reader.

### Statistical analysis

In addition to the microarray analysis, quantitative data are shown as the mean ± SE. Statistical analyses were performed with Prism version 5.0c (GraphPad Software, San Diego, CA). When comparisons between multiple groups were made, analysis of variance with the Student-Newman-Keuls posthoc multiple comparisons test was performed. When comparisons between only 2 groups were made, Student's *t* test was performed. The log-rank (Mantel-Cox) test was used for the survival analysis.

## Results

### DSS-induced colitis alters serum amino acid levels and increases tissue L-arginine uptake

**Figure 1:**
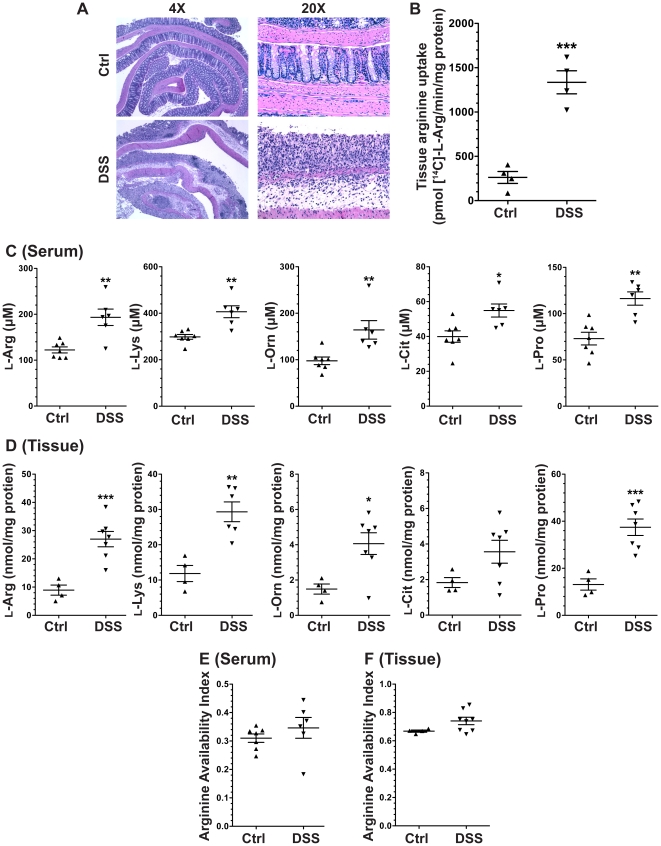
DSS-induced colitis alters serum amino acid levels and increases tissue L-Arg uptake. 7-week-old C57BL/6 mice received 4% DSS in the drinking water or water alone (Ctrl) for 7 days. At sacrifice on day 7, blood was obtained via cardiac puncture. Serum was obtained after 30 min, snap frozen, and the amino acid profile was subsequently assessed by HPLC. The colon was removed and fresh pieces of tissue were obtained to assess the amino acid profile and L-Arg uptake, as described in the Methods. (A) Representative photomicrographs showing presence of colitis after 7 days versus Ctrl. (B) L-Arg uptake in colonic tissue. (C) Serum amino acid levels. (D) Tissue amino acid levels. (E) Serum AAI. (F) Tissue AAI. *n* = 4–8 per group. *p<0.05, **p<0.01, ***p<0.001 vs. control.

After 7 days of exposure to 4% DSS in the drinking water, there was evidence of histologic injury versus control animals. Representative photomicrographs are shown (Figure 1A). There was a significant, 5.1±1.9–fold increase in L-Arg uptake detected in the DSS tissues *ex vivo* (Figure 1B). In the mice treated with DSS, there was an increase in serum L-Arg, L-Lys, L-Orn, L-Pro, and L-Cit concentrations versus untreated control mice (Figure 1C). However, there was not a significant increase in the AAI (Figure 1E), which takes into account the interaction of L-Orn and L-Lys with the L-Arg transporter CAT2. In parallel with the increases in serum amino acids, there were significant increases in tissue L-Arg, L-Lys, L-Orn, and L-Pro concentrations versus control (*p*<0.05), and a modest increase in L-Cit that was not significant (Figure 1D). There was no significant increase in the tissue AAI (Figure 1F). Taken together these data suggest that there are increases in multiple amino acids in acute DSS colitis, but there is no increase in AAI in either serum or tissue, indicating that L-Arg supplementation could be useful particularly because tissue L-Arg uptake is enhanced.

### DSS-induced colitis alters L-Arg metabolic pathway mRNA and protein expression

**Figure 2:**
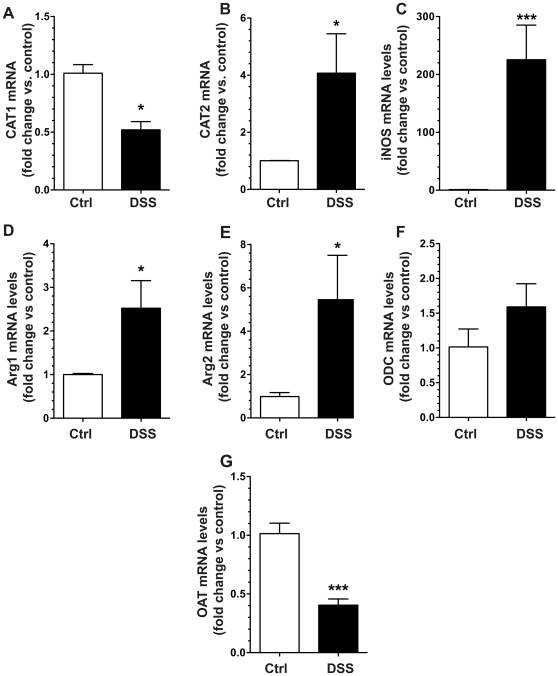
DSS-induced colitis alters mRNA expression of L-Arg metabolic pathway genes. 7-week-old C57BL/6 mice received 4% DSS in the drinking water or water alone (Ctrl) for 7 days. At sacrifice on day 7, the colon was removed and tissues were obtained for RNA isolation and assessment of gene expression by real-time PCR. (A–G) Tissue mRNA expression of L-Arg metabolic pathway genes: L-Arg transporters CAT1 (A) and CAT2 (B), metabolic enzymes iNOS (C), Arg1 (D), and Arg2 (E), as well as the downstream enzymes ODC (F) and OAT (G). *n* = 4–10 per group. *p<0.05, ***p<0.001 vs. control.

Given the alteration in serum and tissue L-Arg concentrations and the increased L-Arg uptake ability of the colitis tissues, we assessed the gene expression of major contributors to the L-Arg metabolic pathway. After 7 days of DSS treatment, there was a significant increase in the mRNA expression of the inducible L-Arg transporter, CAT2, with a significant decrease in CAT1 (Figure 2A and 2B). The mRNA levels of the L-Arg metabolic enzymes iNOS, Arg1 and Arg2 were all significantly increased (Figure 2C–E), while the level of the downstream enzyme, ODC, was modestly, but not significantly increased (Figure 2F). OAT mRNA expression was significantly decreased (Figure 2G).

**Figure 3:**
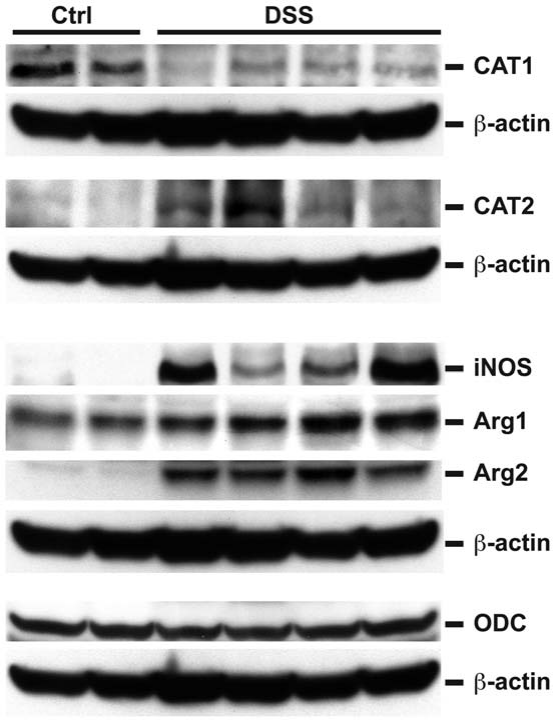
DSS-induced colitis alters L-Arg metabolic pathway protein levels. 7-week-old C57BL/6 mice received 4% DSS in the drinking water or water alone (Ctrl) for 7 days. At sacrifice on day 7, the colon was removed and tissues were obtained for protein isolation and assessment by Western blotting. Each lane represents a different mouse. There were two separate blots that were used, so the appropriate β-actin housekeeping gene control is placed under the blots for the proteins of interest that were performed on those same membranes.

When protein expression was assessed after 7 days of DSS in colon tissues by Western blot analysis (Figure 3), we confirmed the decrease in CAT1, and increases in CAT2, iNOS, and Arg2 when compared to control mice. There was a more modest increase in Arg1 and no change in ODC levels (Figure 3).

### L-Arg supplementation improves clinical parameters in DSS-induced colitis in WT C57BL/6 mice

**Figure 4 pone-0033546-g004:**
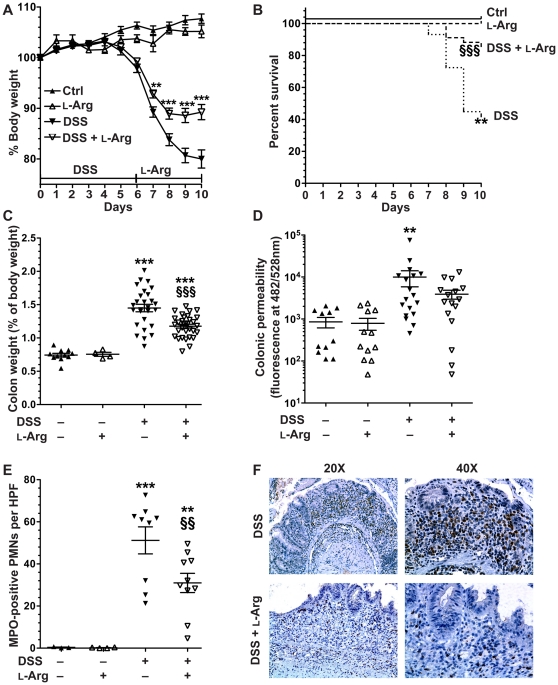
L-arginine improves clinical parameters, mucosal integrity, and PMN inflammatory cell infiltration in DSS-induced colitis. 7-week-old C57BL/6 mice received 4% DSS in the drinking water or water alone (Ctrl) for 6 days, followed by administration of 1% L-Arg in the drinking water, or water alone for 4 days. (A) Body weights of the mice were measured daily and presented as a percentage of their initial body weight. **p<0.01, ***p<0.001 vs. DSS. (B) Survival analysis, with mortality based on >20% body weight loss. **p<0.01 vs. Ctrl. §§§*p*<0.001 vs. DSS. (C) At sacrifice, colons were removed, cleaned, and weighed. The fresh colon weight was determined as a percentage of the total body weight. ***p<0.001 vs. control and §§§*p*<0.001 vs. DSS. In A–C, *n* = 4–11 for controls, *n* = 27–32 for treatments. (D) Prior to sacrifice, mice were anesthetized and given FITC-labeled dextran via rectum. Blood was obtained by cardiac puncture at 2 h. Fluorescence was measured in the serum at 482 nm and 528 nm. In (D), *n* = 4–8 for controls, *n* = 14–16 for treatments. **p<0.01 vs. control. (E) and (F) At sacrifice, colons were removed, cut open longitudinally, cleaned, Swiss-rolled and formalin fixed for future staining with MPO antibody. (E) The number of MPO-positive PMNs per HPF were counted. 20 HPFs per slide were assessed. (F) Representative photomicrographs of MPO staining after DSS treatment alone or DSS followed by L-Arg supplementation. *n* = 4 for controls, *n* = 9–10 for treatments. **p<0.01, ***p<0.001 vs. control. §§*p*<0.01 vs. DSS.

Mice were administered 4% DSS in their drinking water for 6 days followed by 4 days of water alone or 1% L-Arg in the drinking water. We followed this protocol of oral administration based on previous protocols from our laboratory and others [25], [53] demonstrating marked effects on colon pathology when L-Arg is added to the drinking water, and evidence that oral amino acid delivery to humans results in rapid and efficient absorption in the small intestine and thus availability for distribution systemically through the circulation [54], [55]. It should be noted that we could not administer the L-Arg during the DSS treatment, as we found that the combination led to precipitation in the drinking water, rendering the combination unreliable. Mice began losing body weight after 5 days of treatment with DSS and in the DSS only group this weight loss reached 10.76±1.01% (89.24±1.01% of starting body weight) by day 7, 16.19±1.18% (83.81±1.18% of starting body weight) by day 8, 19.31±1.42% (80.69±1.42% of starting body weight) by day 9, and 19.99±1.79% (80.01±1.79% of starting body weight) by day 10 (Figure 4A). In contrast, the body weight loss of mice receiving supplemental L-Arg after exposure to 6 days of DSS was only 7.16±0.78% (92.84±0.78% of starting body weight) by day 7, 11.05±1.09% (88.95±1.09% of starting body weight) by day 8, 11.41±1.39% (88.59±1.39% of starting body weight) by day 9, and 10.74±1.51% (89.26±1.51% of starting body weight) by day 10 (Figure 4A). The abrogation of body weight loss by L-Arg supplementation was significantly different at days 7 (*p*<0.01), and 8, 9, and 10 (*p*<0.001 for each) versus the DSS only mice. Mice not given DSS that received L-Arg alone did not exhibit any increase in their body weight, and in fact they showed a modest decrease compared to the control group receiving water alone (Figure 4A). Therefore, the increase in body weight with L-Arg supplementation of the DSS-treated mice is not attributable to a nonspecific effect on weight gain.

There was also substantially less mortality with L-Arg supplementation (Figure 4B), as survival improved from 41.3% in the DSS group to 85.3% in the DSS+L-Arg group (*p*<0.001). Colon weight as a percentage of total body weight, a gross indicator of inflammation, was markedly increased in the DSS only group (Figure 4C), and this increase was significantly attenuated in the DSS+L-Arg group (*p*<0.001). In addition, the colon weight/colon length ratio, another parameter that quantifies DSS-induced injury, showed a similar increase in the DSS group that was also attenuated in the DSS+L-Arg group (Figure S1).

### L-Arg supplementation abrogates the increased colonic mucosal permeability in response to DSS-induced colitis

Colonic mucosal integrity was assessed by a FITC-dextran permeability assay. There was a significant increase in colonic mucosal permeability after exposure of mice to DSS for 6 days followed by 4 days of water (*p*<0.001), compared to control mice not receiving DSS (Figure 4D). This increase was abrogated in the mice given DSS followed by supplemental L-Arg.

### L-Arg supplementation reduces the MPO-positive cellular infiltrate in DSS-induced colitis

DSS colitis is characterized by infiltration of immune cells in the mucosa and submucosa, epithelial ulceration, and crypt damage [36], [40], [41]. As expected, there was a substantial increase in the colon histologic injury score after treatment with DSS followed by water alone; however, L-Arg supplementation did not lead to a significant difference in the histologic injury score (13.9±1.3 for DSS, and 14.7±1.2 for DSS+L-Arg, data not shown). We therefore assessed the cellular inflammatory infiltrate present in more detail. MPO staining allows for reliable and simple *in situ* quantification of polymorphonuclear cells (PMNs). As shown in Figure 4E and 4F, there was a significant increase in MPO-positive cells in the colon tissue after exposure to DSS compared to control mice (*p*<0.001). Importantly, with DSS followed by L-Arg compared with DSS alone, there was a 39.5±5.5% inhibition in MPO-positive PMNs/HPF (*p*<0.01; Figure 4E).

### Serum L-Arg concentration is no longer increased in a recovery model of DSS-induced injury, while tissue L-Arg and L-Pro remain increased

After 6 days of exposure to 4% DSS in the drinking water followed by 4 days of water there was a modest increase in L-Arg in the serum, but this was not statistically significant, and the small further increase in serum L-Arg with 1% L-Arg in the drinking water was also not significant (Table 1). However, there was a significant increase in serum L-Lys levels in the DSS group (Table 1), which is relevant because L-Lys can function as an endogenous inhibitor of iNOS, as it blocks NO generation via inhibition of L-Arg uptake, as we have demonstrated in macrophages [43] and in colonic epithelial cells (data not shown). Importantly, L-Arg supplementation of mice significantly reduced circulating L-Lys levels (Table 1). However, DSS also led to modest increases in serum L-Orn that were not changed with L-Arg treatment, and for this reason the overall AAI was not increased (Table 1). There were no significant alterations in serum L-Cit or L-Pro levels in the DSS or DSS+L-Arg groups.

**Table 1:** Amino acids in the serum from DSS colitis in the presence or absence of L-Arg.

	Control	Control+l-Arg	DSS	DSS+l-Arg
	*n* = 10	*n* = 8	*n* = 13	*n* = 20
l-Arg	125.0±21.3	119.1±15.8	180.1±26.1	206.2±23.8
l-Lys	309.9±19.6	283.2±15.2	448.7±26.2***	382.1±18.6* §
l-Orn	106.3±20.6	168.3±31.6	203.9±41.2	256.3±53.2
l-Cit	38.9±2.2	40.9±4.0	48.3±4.7	43.6±2.1
l-Pro	75.1±7.1	80.2±4.6	97.9±12.7	94.3±10.3
AAI	0.3±0.1	0.3±0.1	0.3±0.1	0.3±0.1

7-week-old C57BL/6 mice received 4% DSS in the drinking water or water alone for 6 days followed by administration of 1% L-Arg in the drinking water, or water alone for 4 days. At sacrifice on day 10, blood was obtained via cardiac puncture. Amino acids were measured in serum (µmol/Liter) as in Figure 1. *n* = 8–20 per group.

* *p*<0.05,

*** *p*<0.001 vs. control.

§ *p*<0.05 vs. DSS.

When tissue amino acids were measured (Table 2), there was a substantial, 2.7±0.8–fold increase in tissue L-Arg concentration after exposure to DSS versus control tissues (*p*<0.01). There was no enhancement of colon tissue L-Arg levels after oral supplementation. There was a modest increase in tissue AAI with DSS treatment, but this was not significant, most likely due to the increase in L-Lys and L-Orn in some of the mice, even though there was not an overall increase in L-Lys and L-Orn levels. One potentially important finding was that L-Pro was increased in tissues from DSS-treated mice at the 10-day timepoint in this recovery model, as this amino acid has been linked to wound repair due to its role in collagen synthesis [21]. However, L-Arg did not enhance the L-Pro levels, suggesting that L-Arg has additional effects.

**Table 2:** Amino acids in the colonic tissue from DSS colitis in the presence or absence of L-Arg.

	Control	Control+l-Arg	DSS	DSS+l-Arg
	*n* = 10	*n* = 8	*n* = 17	*n* = 14
l-Arg	2.3±0.4	2.6±0.3	6.2±1.1**	5.4±1.3*
l-Lys	5.0±0.9	3.6±0.4	7.3±1.3	5.6±0.9
l-Orn	0.5±0.1	0.8±0.1	0.9±0.2	1.0±0.2
l-Cit	0.7±0.1	1.4±0.3	1.1±0.2	1.0±0.1
l-Pro	2.6±0.5	3.5±0.5	7.0±1.1**	5.3±0.9*
AAI	0.5±0.1	0.6±0.1	0.6±0.1	0.6±0.1

7-week-old C57BL/6 mice received 4% DSS in the drinking water or water alone for 6 days followed by administration of 1% L-Arg in the drinking water, or water alone for 4 days. At sacrifice on day 10, the colon was removed and a fresh piece of tissue was snap frozen. Amino acids were measured in the tissue (µmol/mg protein) as in Figure 1. *n* = 8–17 per group.

* *p*<0.05,

** *p*<0.01 vs. control.

### Sustained induction of CAT2, iNOS, and Arg1, and downregulation of ODC in the DSS injury and repair recovery model

**Figure 5 pone-0033546-g005:**
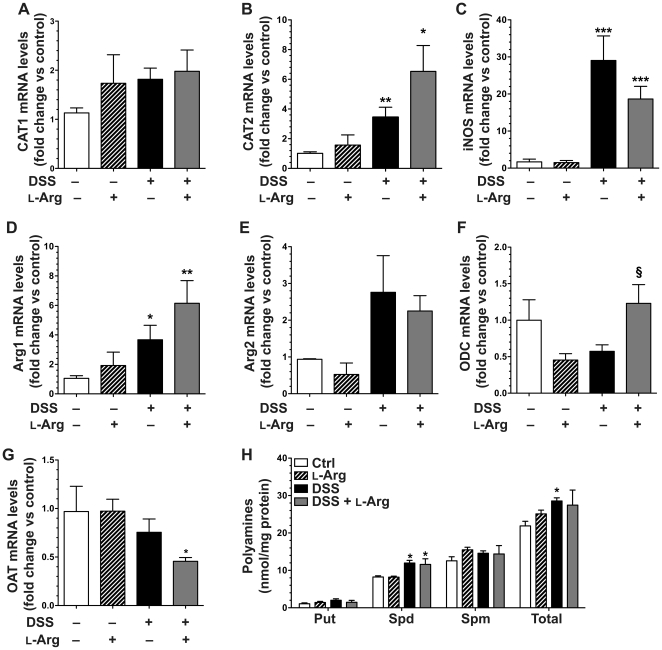
Effect of DSS treatment and L-Arg supplementation on L-Arg metabolic pathways. Mice received 4% DSS ± 1% L-Arg in the drinking water as in Figures 3–4. (A) At sacrifice on day 10, a piece of colon was obtained for RNA isolation or polyamine determination by HPLC. (A–G) Tissue mRNA levels of the genes indicated, as assessed by real-time PCR. (H) Tissue polyamine levels were assessed by HPLC as described in the Methods. *n* = 4–15 per group. *p<0.05, **p<0.01, ***p<0.001 vs. control. §*p*<0.05 vs. DSS.

To further examine L-Arg utilization, we conducted mRNA expression studies of relevant genes after 6 days of DSS followed by recovery with water alone or 1% L-Arg for 4 days. At this timepoint, there was no longer a decrease in levels of CAT1 (Figure 5A) that was observed after 7 days of continuous DSS in Figure 2, and CAT2 expression was still significantly increased with DSS alone and with DSS+L-Arg, where a modest further increase was observed (Figure 5B). iNOS (Figure 5C) and Arg1 (Figure 5D) mRNA levels both continued to be upregulated with DSS at the 10-day timepoint in the absence or presence of L-Arg, whereas the small increase in Arg2 levels with DSS was not significant compared to control mice (Figure 5E). The expression of the downstream enzyme ODC was somewhat reduced with DSS, and L-Arg supplementation returned this back to control levels (Figure 5F). Finally, there was a small decrease in OAT expression with DSS alone, but surprisingly, in mice receiving DSS+L-Arg there was a significant reduction in OAT levels (Figure 5G). Because polyamines have been implicated in wound repair, and we previously found an increase in polyamines with L-Arg treatment in the *C. rodentium* model [25], we measured polyamine levels in colonic tissues. There was a significant increase in spermidine levels with DSS treatment with small, not significant increases in putrescine and spermine, with an overall significant increase in total polyamines (Figure 5H). However, L-Arg supplementation did not have any effects on the individual or total polyamine levels, despite the clinical improvement in these mice. Taken together with the amino acid data, these findings suggest that the clinical improvement of the mice in response to L-Arg is not likely due to enhanced generation of polyamines by ODC or L-Pro by OAT, but rather by other aspects of L-Arg metabolism.

### Altered tissue chemokine and cytokine levels in DSS return to baseline with L-Arg supplementation

**Figure 6 pone-0033546-g006:**
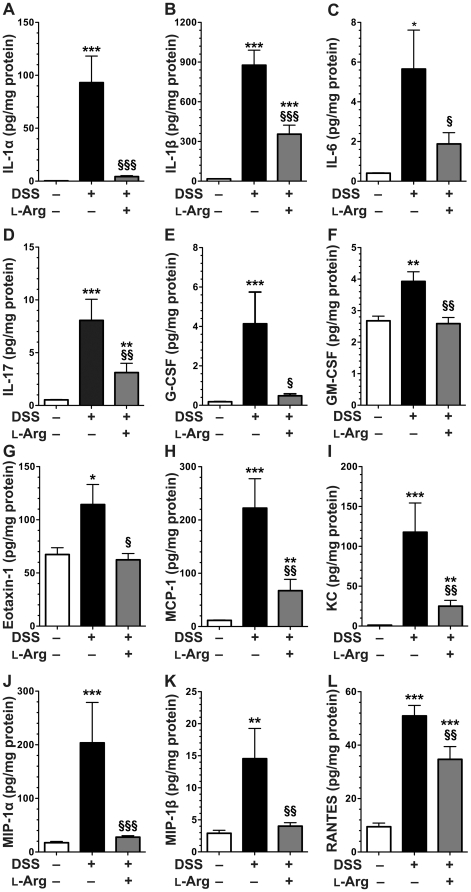
Altered tissue chemokine/cytokine levels in DSS return to baseline with L-Arg supplementation. Mice received 4% DSS ± 1% L-Arg in the drinking water as in Figures 3–5. (A–L) At sacrifice on day 10, the colons were harvested and samples from the proximal and distal end were obtained and snap frozen. They were subsequently lysed in RIPA buffer. Tissue lysates were assessed by BioPlex® multiplex bead-based antibody detection using Luminex technology. Protein concentrations in the lysates were determined using BCA assay for normalization. *n* = 5 mice in control group, *n* = 7–8 mice in treatment groups. *p<0.05, **p<0.01, ***p<0.001 vs. control. §*p*<0.05, §§*p*<0.01, §§§*p*<0.001 vs. DSS.

To provide further insight into the immunologic effects of L-Arg treatment that might underlie the clinical improvement in the mice, we conducted analysis of cytokines and chemokines in colon tissue lysates by Luminex assay (Figure 6), using a 23 analyte kit. For IL-12p70, all levels were below the limit of detection. For the remaining analytes, exposure to DSS resulted in a significant increase in the proinflammatory cytokines IL-1α, IL-1β, IL-6, IL-17, G-CSF, GM-CSF, and the proinflammatory chemokines eotaxin-1, KC, MCP-1, MIP-1α, MIP-1β, and RANTES (Figure 6). Importantly, each of these increases with DSS were significantly decreased by L-Arg supplementation (Figure 6). IL-4 was also decreased in the DSS+L-Arg group when compared to DSS alone, but the level of IL-4 was below the limit of detection in control mice, making these data difficult to fully assess. IL-2 and IL-12 p40 were significantly increased after exposure to DSS, but were not significantly altered by L-Arg supplementation (data not shown). Cytokines not altered by exposure to DSS in this assay included IL-3, IL-5, IL-9, IL-10, IL-13, TNF-α, and INF-γ (data not shown).

### Analysis by microarray reveals multiple differentially expressed genes in DSS-induced colitis which are altered with L-Arg supplementation

**Figure 7 pone-0033546-g007:**
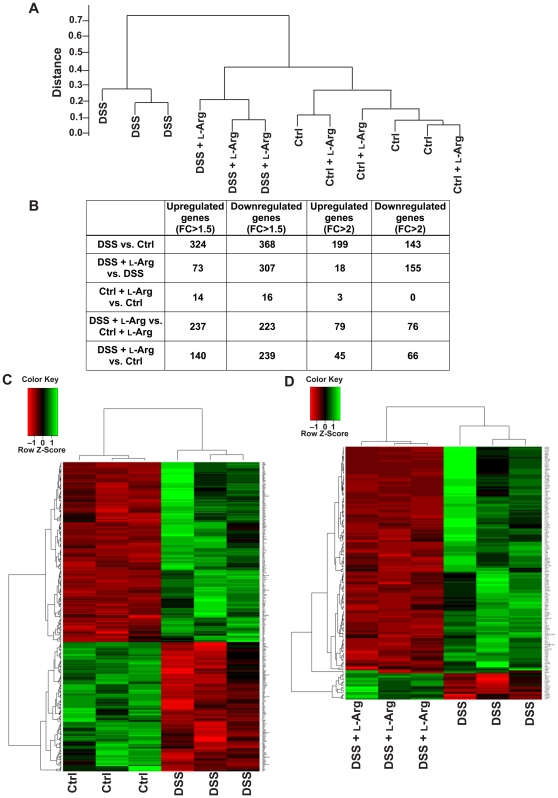
Tissue microarray assessment identifying multiple differentially expressed genes after exposure to DSS. Mice received treatments as in Figures 3–6. There were four groups, namely control (Ctrl), receiving water alone; control+L-Arg, receiving water for 6 days and L-Arg for 4 days; DSS, receiving DSS for 6 days and water for 4 days; and DSS+L-Arg, receiving DSS for 6 days and L-Arg for 4 days. At sacrifice, a piece of colon was obtained and placed in RNAlater®. Three samples from each of the four groups were submitted for microarray assessment. (A) Hierarchical clustering of the samples based on >16,000 protein-coding genes. The 12 samples were clustered by the expression values of the probes to examine the inter- and intra-groups relationship of the samples. Note that the DSS group was markedly distinct from the Ctrl or the Ctrl+L-Arg groups, and the DSS+L-Arg group segregated closely with the control groups not receiving DSS, rather than with the DSS group. (B) Table of the number of differentially expressed genes (DEGs) based on *p* value<0.01 and fold change (FC) of >1.5 or >2. (C) Heatmap of the DEGs between the Ctrl and DSS groups. (D) Heatmap of the DEGs between the DSS and DSS+L-Arg groups. Green is increased expression and red is decreased expression.

Having detected substantial differences in cytokines and chemokines, despite a lack of difference in histology, we sought to establish the degree of difference between our experimental groups by a more comprehensive genetic approach, namely comparative transcriptome analysis. Hierarchical clustering was conducted for the same four mouse groups in the 10-day model used in Figures 4 and 5. There were 3 mice in each group. When we compared expression values in the 12 samples, the DSS group was markedly distinct from the control or the control+L-Arg groups. Importantly, the DSS+L-Arg group segregated closely with the two control groups not receiving DSS, rather than with the DSS group (Figure 7A). We then assessed the differentially expressed genes (DEGs) in different combinations of two group comparisons, examining those with a *p* value<0.01 and a fold change of >1.5 or >2 (Figure 7B). We chose the more stringent criteria with the fold change >2 to proceed with further analysis. There were 342 DEGs with DSS versus control, but only 59 of these genes were altered in DSS+L-Arg versus control (Figure S2). The detailed patterns are presented by heatmaps, which clearly demonstrate marked changes with DSS versus control (Figure 7C) and in DSS+L-Arg versus DSS alone (Figure 7D).

**Figure 8 pone-0033546-g008:**
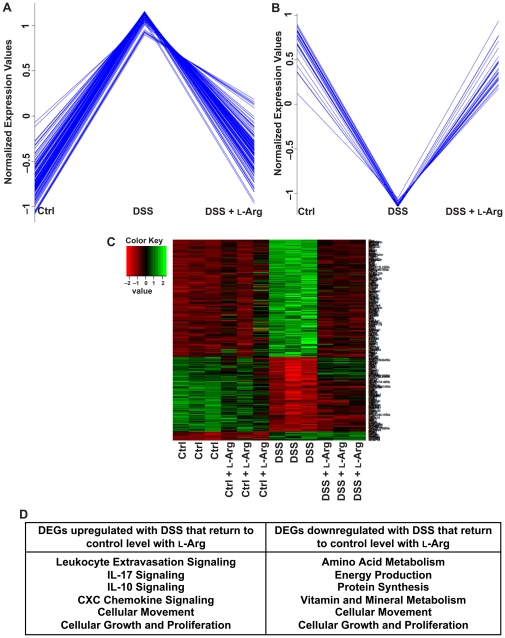
Multiple pathways altered after exposure to DSS return to baseline with L-Arg supplementation. Pathway analysis based on DEGs. (A) 132 genes were found to be upregulated in response to DSS that were subsequently downregulated to control levels after L-Arg supplementation. (B) 27 genes were downregulated in response to DSS and returned to control levels after L-Arg supplementation. (C) Heatmap of the DEGs based on the expression pattern of increased expression after exposure to DSS and decreased expression following L-Arg supplementation. (D) Table showing examples of biologic and signaling pathways, derived from Ingenuity Pathway Analysis, that were found to be affected by these altered gene expression patterns.

We performed a more in-depth analysis to identify the pattern of alteration in gene expression related to the treatments given to the mice. With hierarchical clustering using a Pearson correlation, we specifically identified 132 genes upregulated in response to DSS that were downregulated to control levels after L-Arg supplementation (Figure 8A). There were also 27 genes that were downregulated with DSS that returned to control levels in the DSS+L-Arg group (Figure 8B). The detailed expression pattern of genes that were altered by DSS and then affected by L-Arg supplementation is presented in a specific heatmap (Figure 8C). As these relationships cannot be directly accessed in the GEO database, Tables S1 and S2 with each gene, its fold change, and *p* value for the two detailed expression patterns are included in the supplemental information.

The pathways enriched with DEGs between DSS+L-Arg and DSS alone included leukocyte extravasation; IL-17, IL-10, and CXC chemokine signaling pathways; cell migration; and cell proliferation (Figure 8D). Also related to our findings of clinical improvement with L-Arg there was a restoration to control levels of DEGs associated with amino acid metabolism, protein synthesis, and energy production.

We then used real-time PCR to confirm results obtained from the microarray. Four gene targets from Figure 8A were assessed in the same RNA samples submitted for microarray analysis. The same expression patterns were seen in MMP-3, MMP-10, MMP-13, and IL-16 (Figure S3).

### L-Arg supplementation alters migration, but not proliferation in isolated colonic epithelial cells from DSS-induced colitis

**Figure 9:**
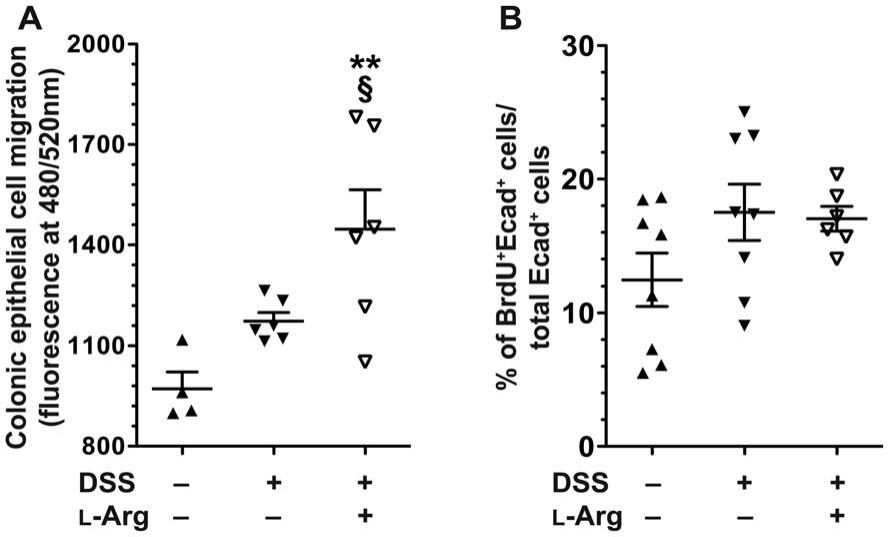
Epithelial cell migration but not proliferation is altered in DSS colitis ± L-Arg. Mice received 4% DSS ± 1% L-Arg in the drinking water as in Figures 3–8. On the day of sacrifice, colons were removed, and epithelial cells were isolated as described in the Methods. (A) Migration assay, performed on freshly isolated CECs, as described in the Methods. (B) On the day of sacrifice, mice were given an i.p. injection of BrdU, CECs were isolated, and stained for BrdU and E-cadherin as described in the Methods. Double-positive cells were detected by flow cytometry. *n* = 4–8 for controls, *n* = 6–8 for treatments. **p<0.01 vs. control. §*p*<0.05 vs. DSS.

Having noted DEGs related to both cellular movement and growth/proliferation, we performed specific analyses of these events in epithelial cells from colitis tissues. Freshly isolated CECs from mice in the 10-day model were assessed in a migration assay (Figure 9A). This revealed a modest increase in cell migration after exposure to DSS alone, which was not statistically significant compared to control mice. However, there was a further increase in cell migration with L-Arg supplementation after DSS treatment that was significant compared to both the control (*p*<0.01) and DSS only (*p*<0.05) groups (Figure 9A). We also assessed the presence of epithelial cell proliferation by BrdU staining (Figure 9B). There was no significant increase in BrdU^+^/E-cadherin^+^ cells after exposure to DSS or to DSS followed by L-Arg supplementation, when compared to control mice (Figure 9B). Taken together, these data indicate that enhancement of cell migration, rather than cell proliferation, may contribute to the beneficial effects of L-Arg during the recovery phase of DSS colitis, as cell migration is an important component of epithelial wound repair.

### Loss of the clinical benefits of L-Arg supplementation in DSS colitis in mice with iNOS deletion

**Figure 10:**
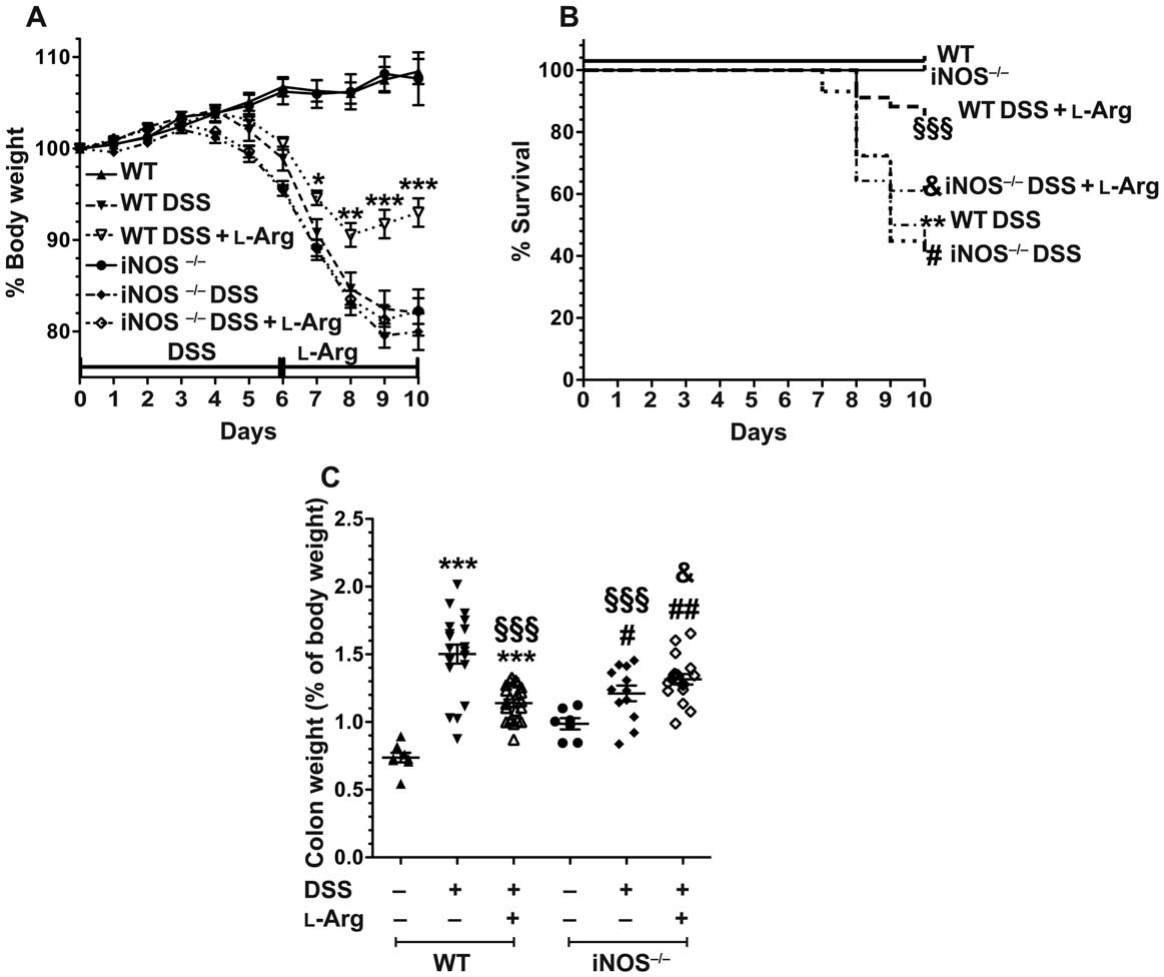
Benefits of L-Arg supplementation in DSS-induced colitis are lost in iNOS^−/−^ mice. 7-week-old WT or iNOS^−/−^ C57BL/6 mice received 4% DSS ± 1% L-Arg in the drinking water as in Figures 3–9. (A) Body weights of the mice were measured daily and are presented as a percentage of their initial body weight. *p<0.05, **p<0.01, ***p<0.001 vs. WT DSS. (B) Survival analysis, as in Figure 3. **p<0.01 vs. WT Ctrl; §§§*p*<0.001 vs. WT DSS; **#** *p*<0.05 vs. iNOS^−/−^ Ctrl; &*p*<0.05 vs. WT DSS L-Arg. (C) At sacrifice on day 10, colon weight as a percentage of the total body weight was determined as in Figure 3. ***p<0.001 vs. WT Ctrl; §§§*p*<0.001 vs. WT DSS; **#** *p*<0.05, **##** *p*<0.001 vs. iNOS^−/−^ Ctrl; &*p*<0.05 vs. WT DSS+L-Arg. In (A–C), *n* = 7–8 for controls, *n* = 12–22 for treatments.

Because there was no demonstrable effect of L-Arg on ODC/polyamines or OAT/L-Pro, we focused on the other pathway of L-Arg utilization, namely iNOS. We compared WT and iNOS^−/−^ mice that were treated with 4% DSS for 6 days followed by 4 days of water alone or 1% L-Arg. As shown previously in Figure 3, WT mice began losing body weight after 5 days of treatment with DSS and continued to lose weight during the recovery period after discontinuation of DSS on day 6 (Figure 10A). The iNOS^−/−^ mice exposed to DSS exhibited the same pattern of body weight loss as the WT mice out to the 10-day timepoint (Figure 10A). However, the marked attenuation of body weight loss with L-Arg treatment in the WT mice was completely lost in the iNOS^−/−^ mice. While in the WT group the abrogation of body weight loss by L-Arg supplementation was significantly different at days 7, 8, 9, and 10 versus the mice receiving water alone after DSS, there was no effect of L-Arg supplementation in the iNOS^−/−^ mice, such that the mice receiving L-Arg after DSS overlapped with those that did not (Figure 10A). As shown in Figure 10B, there was a significant survival benefit with L-Arg supplementation in the WT mice (consistent with our previous results in Figure 3B), but there was no significant enhancement of survival with L-Arg in iNOS^−/−^ mice compared to WT or iNOS^−/−^ mice exposed to DSS alone. Similarly, the reduction in colon weight with L-Arg treatment of WT mice receiving DSS was also eliminated in the iNOS^−/−^ mice (Figure 10C). Taken together, these data indicate that the clinical amelioration of colitis with L-Arg does not occur in iNOS^−/−^ mice, thus implicating iNOS in the beneficial effects of L-Arg.

## Discussion

The objective of this study was to determine whether L-Arg supplementation could abrogate disease in a murine model of epithelial injury and colitis and thus could have potential as a therapy in human IBD. The DSS model we used has been compared to human UC due to the epithelial destruction and presence of ulcers confined to the mucosa [36]. Moreover, the DSS model provides an opportunity to examine treatments during the recovery phase after withdrawal of the injurious agent, as we performed in the current study. We have now demonstrated that L-Arg supplementation is associated with improvement in clinical measures of colitis, including body weight loss and survival; morphological parameters such as colon weight; inflammatory markers, including proinflammatory cytokine and chemokine production; and epithelial cell migration in response to injury.

We have previously reported that L-Arg supplementation is beneficial in *C. rodentium* infection, but in that model serum L-Arg levels were depleted, whereas in the current work we found that serum L-Arg levels were increased in acute DSS colitis after 7 days of continuous exposure. Similarly, we detected that in human UC patients with severe colitis there were increased serum L-Arg concentrations compared to normal controls [27]. However, relative arginine availability, as calculated by the AAI, was not increased in these human subjects due to the concomitant increase in L-Orn and L-Lys that can act as competitive inhibitors for L-Arg transport by CAT1 or CAT2 at the cell membrane [6], [10], [11], [26]. These human serum findings are comparable to what we observed in the current study in the acute DSS model at the 7-day timepoint, and in the 10-day injury and repair model we detected no increase in the serum AAI. Importantly, there was an increase in tissue L-Arg in the 10-day model, but the tissue AAI was not increased. Interestingly, while there was a beneficial effect of L-Arg treatment, this did not further increase tissue L-Arg levels, suggesting that the additional L-Arg was metabolized. To our knowledge, tissue L-Arg levels have not previously been directly quantified in colon tissues from either animals or humans. We also demonstrate for the first time that the L-Arg transporter, CAT2, is upregulated in DSS colitis tissues in both the acute 7-day model and in the 10-day injury and repair model, while CAT1 is decreased in the first model and not altered in the second. These data indicate that CAT2 is important in colitis, which is supported by our data showing an increase in L-Arg uptake and in tissue L-Arg levels with DSS colitis. Another point raised by the increased L-Arg uptake in colitis tissues is that this occurs despite the fact that DSS induces epithelial injury. This increase may be due to increased L-Arg transport in intact epithelium adjacent to areas of ulceration, as epithelial destruction is only partial in our model [36], and/or uptake by other cells, especially infiltrating macrophages, which we have shown to exhibit increased uptake of L-Arg during mucosal inflammation [43].

Another intriguing finding in our study was a lack of improvement in histologic injury score with L-Arg treatment, despite substantial amelioration of mortality, body weight loss, and colon weight. When we tested the effect of L-Arg at a later time point, namely 6 days of DSS followed by 7 days of L-Arg, there was also improvement in clinical parameters without improvement in histology scores (data not shown). Also, when we tested a higher concentration of L-Arg (5% in the drinking water), the mice did not tolerate this in the control group, and the experiment could not be continued. Similarly, we also observed that in the *C. rodentium* model there was significant improvement in clinical parameters without a substantial effect on histologic score [25].

However, when we specifically analyzed the MPO staining as a marker of PMN infiltration, we found that the marked increase with DSS was significantly attenuated with L-Arg treatment. This is meaningful because neutrophil accumulation has been shown to have an important role in DSS-induced colitis, since the disease can be attenuated when anti-neutrophil serum is administered [56]. These findings suggest that conventional scoring of inflammation by hematoxylin and eosin staining may lack sensitivity to detect differences in specific inflammatory cell infiltrates in this model. Consistent with our MPO data, L-Arg attenuated the increased tissue levels of the PMN growth factors G-CSF and GM-CSF, and several chemokines linked to PMN chemoattraction, such as KC and MIP-1α, in colonic tissue from DSS-treated mice. In particular, the chemokine data provides support for L-Arg as an anti-inflammatory agent, since chemokine expression is recognized as a key early step in the pathophysiology of inflammatory responses to colonic pathogens and has been directly linked to the pathophysiology of human IBD [40]. Our findings that L-Arg also effectively reduced other proinflammatory mediators, such as the innate immune cell products IL-1α, IL-1β, and IL-6, as well as the prototype Th17 cytokine, IL-17, suggest that it may be broadly beneficial in colitis. The effect on IL-17 is particularly noteworthy, as the Th17 subset of lymphocytes is now considered to play an important role in the immunopathogenesis of IBD [57], [58].

An interesting question is whether induction of DSS colitis results in a systemic immune response. To this end, we have conducted Luminex-based profiling of 14 analytes that included a selection of Th1, Th2/Treg, Th17 cytokines, as well as chemokines. We found that 10 of these were significantly increased in colitis tissues in acute colitis (7-day model), namely IFN-γ, TNF-α, IL-1β, IL-6, IL-10, IL-17, MIP-1α, KC, MCP-1, and MIP-1β, demonstrating a mixed picture of Th1/Th17 cytokines plus pro-inflammatory chemokines; when assessed in serum, 6 of these were also increased, specifically IFN-γ, TNF-α, IL-1β, IL-6, KC and MCP-1, suggesting a Th1 bias, and a systemic pro-inflammatory effect (data not shown). Because of the systemic IFN-γ response, we conducted exploratory studies of this prototype Th1 cytokine in splenocytes from mice exposed to DSS for 6 days plus 4 days of recovery, using a standard ex vivo model of culture with anti-CD3 and anti-CD28 followed by activation with phorbol 12-myristate 13-acetate (PMA) and ionomycin. When assessed by flow cytometry, there was a marked increase in intracellular IFN-γ in CD4^+^ lymphocytes from mice receiving DSS that was decreased by 48% in cells from mice receiving L-Arg during the recovery period (data not shown). Taken together, these additional findings indicate further studies of the systemic immune response could be a fruitful area of investigation to enhance the development of rational immunomodulatory therapeutic strategies.

Given the improvement of multiple clinical parameters after L-Arg supplementation, we assessed the potential effect on epithelial migration, proliferation, and mucosal integrity. Our *ex vivo* assessment of freshly isolated CECs showed a modest increase in epithelial migration after exposure to DSS, and a significant further increase with L-Arg supplementation. These data are consistent with unpublished data from our lab using an *in vitro* wound restitution assay in a colonic epithelial cell line demonstrating that L-Arg supplementation improves epithelial wound repair. Polyamines, which are a product of L-Arg metabolism, have been shown to play a role in intestinal cell migration [18], [59]. However, we found that while there was a significant increase in the level of spermidine and the total polyamines in response to DSS exposure, there was not a significant effect of L-Arg supplementation. There was also a decrease in OAT expression, and a modest decrease in L-Pro levels with L-Arg treatment in DSS colitis, also suggesting that L-Arg was not exerting its effects via L-Orn metabolism. We plan to investigate these issues with testing of other amino acids as supplements in the DSS model in our future studies.

Importantly, there was a sustained increase in CAT2 and iNOS mRNA levels in the 10-day model. Moreover, we detected a 7.6–fold increase in iNOS expression with DSS versus control in our microarray analysis (*p*<0.01) that was not decreased with L-Arg (data not shown). There is evidence that supports both protective and deleterious roles of iNOS in colonic inflammation including in the DSS model [23], [25], [60], [61]. In our current studies investigating the role of iNOS in the L-Arg effect, we found that iNOS^−/−^ mice no longer exhibited the clinical benefits of L-Arg supplementation in the DSS model. In our previous report, *C. rodentium* colitis was improved by iNOS deletion and L-Arg treatment of iNOS^−/−^ mice led to an additive benefit [25], suggesting that in that colonic infection overproduction of NO can be deleterious, and utilization of L-Arg by alternative pathways is beneficial. However, in the present studies, utilization of L-Arg by the arginase to ODC/OAT pathway is not implicated in the improved response to DSS injury. Consistent with this conclusion, our unpublished data show that mice with heterozygous deletion of ODC exhibit a reduction in colitis induced by DSS when compared to control mice.

Our data raise the question as to how iNOS may mediate the beneficial effects of L-Arg. In some experimental systems there have been reports of NO enhancing epithelial wound repair [18], [62], [63], [64]. Additionally, L-Arg supplementation has been shown to be beneficial in cardiac ischemia, and notably, this has been attributed to possible scavenging of oxyradicals by NO [65]. There also could be inhibitory effects of iNOS on immune cell responses. We have reported that mice infected with the gastric pathogen *Helicobacter pylori* exhibit inverse relationship between gastric macrophage iNOS levels and gastric inflammation [43]. We also showed that mice with deletion of CAT2, which express decreased iNOS in gastric macrophages, exhibit increased gastric inflammation in this model [48].

Our microarray data provide strong evidence that L-Arg treatment restores aberrant expression of numerous genes that are upregulated or downregulated with DSS colitis. The specific implication of leukocyte extravasation may be related to the inhibitory effect of iNOS on adhesion of leukocytes to endothelial cells [66], and could have therapeutic relevance considering findings that intestinal microvascular endothelial cells from IBD patients exhibit reduced iNOS expression and a hyperadhesive phenotype [67]. The additional pathways implicated in the microarray studies represent specific targets for additional future studies, especially genes implicated in cell migration, including the matrix metalloproteinases, some of which we confirmed in Figure S3.

In summary, the present study provides *in vivo* evidence that L-Arg can improve clinical and biochemical parameters in a murine colitis model of injury and repair with similarities to human UC. The substantial effects of L-Arg on inflammatory mediators and on signaling pathways suggests the potential for broad applicability in other models, such as chronic models of colitis and colitis-associated carcinoma.

## Supporting Information

Figure S1 **L-Arg improves colon weight to length ratio in DSS colitis.** 7-week-old C57BL/6 mice received 4% DSS ± 1% L-Arg in the drinking water as in Figure 3. The fresh colon weight in relation to colon length is shown for the same mice in Figure 3C. ***p<0.001 vs. control and §§§*p*<0.001 vs. DSS. *n* = 4–11 for controls, *n* = 27–32 for treatments.
(TIF)

Figure S2 **Venn diagram for the DEGs identified from the DSS vs. Control, DSS+L-Arg vs. Control, and DSS+L-Arg vs. DSS groups.** The red, blue and green circles represent the number of DEGs for the group comparisons indicated. The colored numbers within the circles represent the number of DEGs unique to that comparison. The black numbers in the intersecting regions represent genes that exhibit shared differential expression between the overlapping groups.
(TIF)

Figure S3 **Confirmation of microarray gene expression data.** 7-week-old C57BL/6 mice received 4% DSS ± 1% L-Arg in the drinking water as in Figures 3–9. At sacrifice on day 10, the colon was removed and a fresh piece of tissue was obtained for RNA isolation. Confirmation of the gene expression values by real-time PCR was performed on the same RNA samples that were used for the microarray analysis. (A–D) MMP-3, MMP-10, MMP-13 and IL-6 followed the same gene expression pattern as in the microarray. *p<0.05, **p<0.01, ***p<0.001 vs. control. §§*p*<0.01, §§§*p*<0.001 vs. DSS.
(TIF)

Table S1 **Expression values for genes following the pattern of increased in DSS and normalized with L-Arg treatment.** Expression levels, fold changes, and *p* values are shown.
(XLS)

Table S2 **Expression values for genes following the pattern of decreased in DSS and normalized with L-Arg treatment.** Expression levels, fold changes, and *p* values are shown.
(XLS)

Methods S1 **Detailed methods for assessment of RNA quality and for transcriptome analysis by microarray.**
(DOC)
