# Supplementary figures and images for: L-arginine Supplementation Improves Responses to Injury and Inflammation in Dextran Sulfate Sodium Colitis
Source: PLoS One. 2012 Mar 12;7(3):e33546. doi: 10.1371/journal.pone.0033546 (PMC3299802; doi:10.1371/journal.pone.0033546)

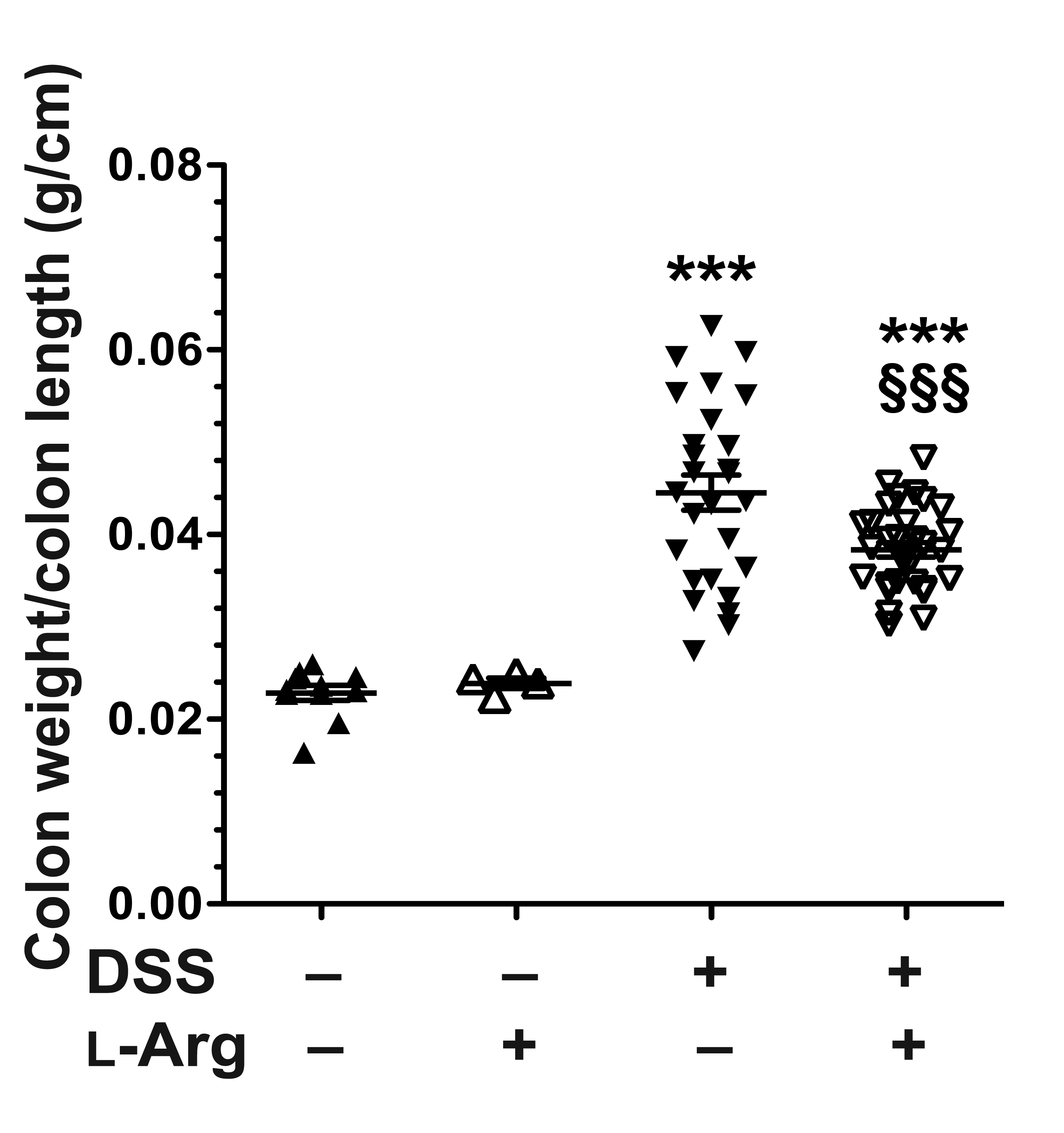

Supplement: Figure S1 — L-Arg improves colon weight to length ratio in DSS colitis. 7-week-old C57BL/6 mice received 4% DSS ± 1% L-Arg in the drinking water as in Figure 3. The fresh colon weight in relation to colon length is shown for the same mice in Figure 3C. ***p<0.001 vs. control and §§§p<0.001 vs. DSS. n = 4–11 for controls, n = 27–32 for treatments. (TIF) [file pone.0033546.s001.tif]

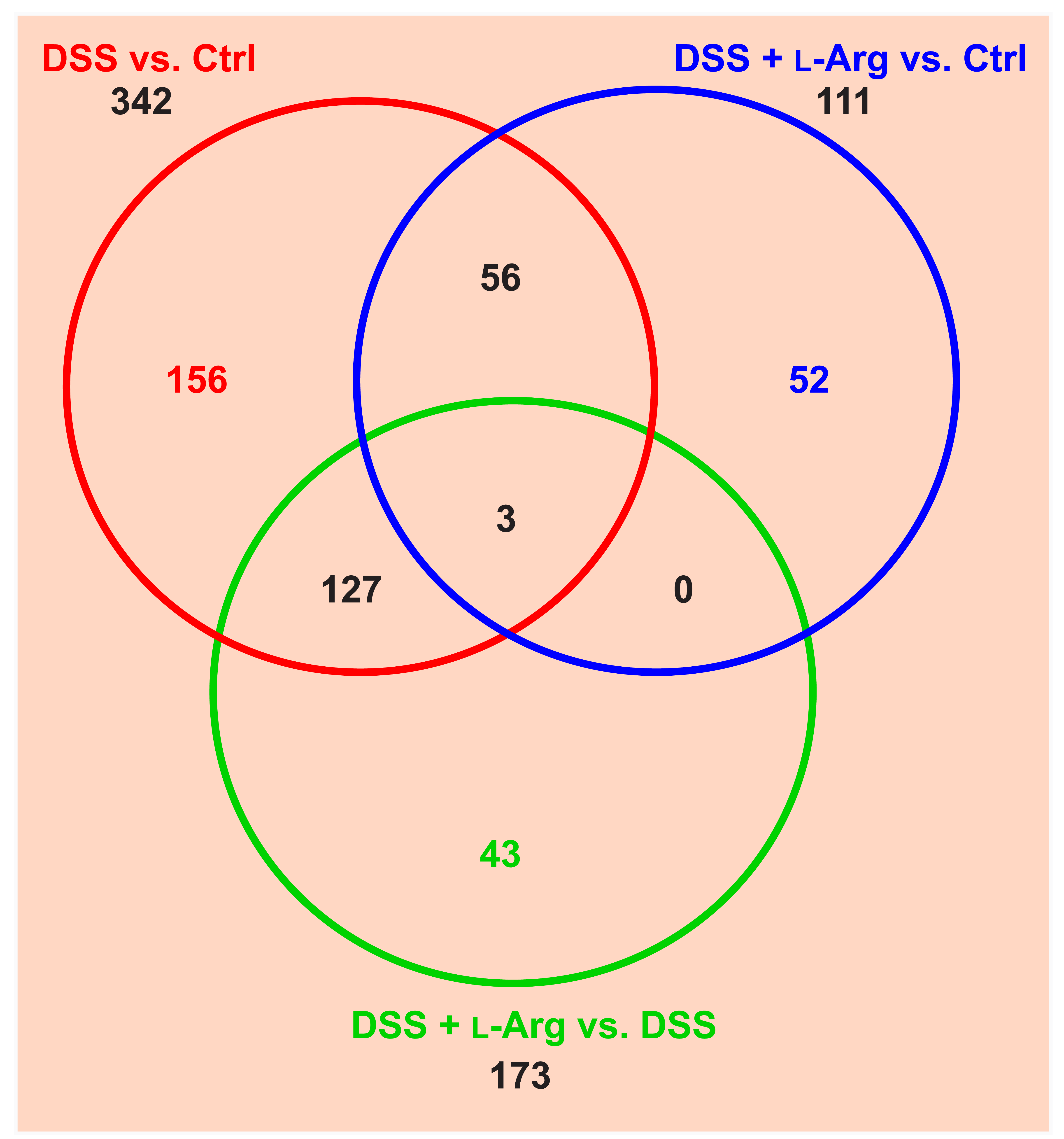

Supplement: Figure S2 — Venn diagram for the DEGs identified from the DSS vs. Control, DSS+L-Arg vs. Control, and DSS+L-Arg vs. DSS groups. The red, blue and green circles represent the number of DEGs for the group comparisons indicated. The colored numbers within the circles represent the number of DEGs unique to that comparison. The black numbers in the intersecting regions represent genes that exhibit shared differential expression between the overlapping groups. (TIF) [file pone.0033546.s002.tif]

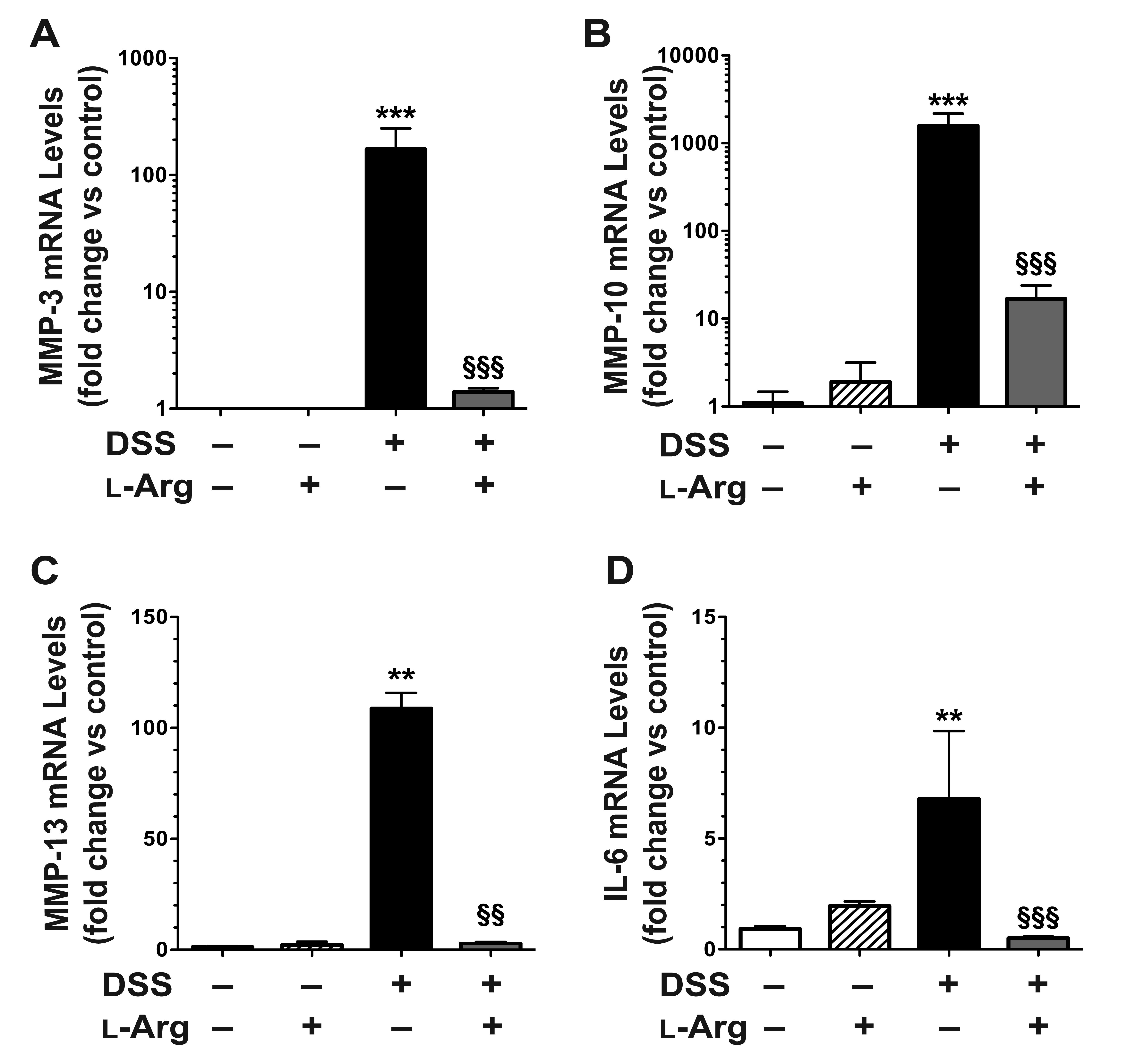

Supplement: Figure S3 — Confirmation of microarray gene expression data. 7-week-old C57BL/6 mice received 4% DSS ± 1% L-Arg in the drinking water as in Figures 3– 9. At sacrifice on day 10, the colon was removed and a fresh piece of tissue was obtained for RNA isolation. Confirmation of the gene expression values by real-time PCR was performed on the same RNA samples that were used for the microarray analysis. (A–D) MMP-3, MMP-10, MMP-13 and IL-6 followed the same gene expression pattern as in the microarray. *p<0.05, **p<0.01, ***p<0.001 vs. control. §§p<0.01, §§§p<0.001 vs. DSS. (TIF) [file pone.0033546.s003.tif]
