# Supplementary material for: L-arginine Supplementation Improves Responses to Injury and Inflammation in Dextran Sulfate Sodium Colitis
Source: PLoS One. 2012 Mar 12;7(3):e33546. doi: 10.1371/journal.pone.0033546 (PMC3299802; doi:10.1371/journal.pone.0033546)
Supplement: Methods S1 — Detailed methods for assessment of RNA quality and for transcriptome analysis by microarray. (DOC) [file pone.0033546.s006.doc]

**Supplemental Methods**

**Method S1.**

**Detailed microarray methods**

RNA samples were prepared from colon tissues as described above and submitted to the Vanderbilt Genomics Sciences Resource where they underwent quality analysis as follows. The sample was quantified in 10 mM Tris on the [Nanodrop ND-1000 spectrophotometer](http://www.nanodrop.com/nd-1000-overview.html) (Thermo Scientific, Willmington, DE) according to the manufacturer’s protocols. In addition to concentration, this instrument reports the 260/280 absorption ratio, which can be an important indicator of hidden contaminants. Each sample was then analyzed for integrity using the [Agilent 2100 Bioanalyzer](http://www.chem.agilent.com/Scripts/PDS.asp?lPage=51) (Agilent Technologies, Santa Clara, CA) according to the manufacturer’s protocols. This instrument produces an electropherogram that allows detection of contaminants or degradation, and allows calculation of the 28s:18s ratio. The Agilent 2100 Bioanalyzer assigns an integrity score, the RNA Integrity Number (RIN), from 1 to 10. For microarray work, it is recommended that all samples have a minimum score of 7.0, and all of our samples had a RIN of > 9.

Affymetrix Whole Transcript Sense reactions, using the Ambion Whole Transcript Reaction kit (catalogue number 4411974, Santa Clara, CA), were assembled and run following the manufacturer’s protocol. Briefly, a sample mix of 100 ng total RNA, and poly-A spike in controls (included in Affymetrix Whole Transcript Terminal Labeling Kit, catalogue number 901524), were brought to a 5 μl final volume with nuclease free H_2_O. First Strand Synthesis Master mix was added to the sample mix and incubated at 25ºC for 1 h, followed by incubation at 42ºC for 1 h. Incubations were followed by cooling to 4ºC for 2 min. Subsequently, Second Strand Synthesis Master mix was added to the sample mix, and incubated at 16ºC for 1 h. The reaction was heat inactivated by a 10 min incubation at 65ºC. The resulting cDNA was then processed in a 16 h *in vitro* transcription reaction to generate cRNA. The cRNA was used in a first strand synthesis reaction to generate a target of the correct sense for hybridization to the Affymetrix Gene and Exon array. The Second Cycle cDNA synthesis reaction was set up with 10 μg cRNA, random hexamer, and first strand synthesis reagents to make cDNA. The cDNA target was then treated with Rnase H to remove template RNA and purified over a kit-supplied column, or Agencourt SPRI beads, following the manufacturer’s protocol (RNAClean). A total of 5.5 μg of the clean cDNA target was then enzymatically fragmented and end-labeled using the Affymetrix kit reagents. The cRNA, cDNA, and fragmented and end-labeled target were assessed by Agilent bioanalysis to insure that the amplified target met the recommended smear range, and that fragmentation and end-labeling were complete. For both Exon and Gene arrays, the requisite amount of target was then added to the hybridization cocktail to give a final target concentration of 25 ng/μl in the hybridization cocktail, per the Affymetrix specifications. The target in the hybridization cocktail was heat denatured at 99ºC for 5 min, cooled to 45ºC for 5 min, centrifuged and then loaded on the Affymetrix cartridge array for a hybridization period of 16 h at 45ºC, with rotation of 60 rpm in the Affymetrix Model 645 Hybridization Oven. A total of 2 μg of fragmented, labeled target was hybridized to the array. The cartridge array was washed and stained per standard Affymetrix Protocols using Affymetrix Hybridization Wash and Stain kit reagents on the Affymetrix Fluidics Station 450, using script FS450_0007 for Gene Arrays. After washing and staining, the array was scanned in an Affymetrix 7G plus scanner using AGCC v. 1.1 and then analyzed with Affymetrix Expression Console v. 1.1 using an RMA normalization algorithm producing log base 2 results.
